# Effect of *Andrographis paniculata* supplementation during the transition period on colostrum yield, immunoglobulin G, and postpartum complications in multiparous sows during tropical summer

**DOI:** 10.5713/ab.23.0190

**Published:** 2024-01-20

**Authors:** Padet Tummaruk, Kankawee Petchsangharn, Kanyakon Shayutapong, Thanwarat Wisetsiri, Patcharin Krimtum, Sidthipong Kaewkaen, Preechaphon Taechamaeteekul, Natchanon Dumniem, Junpen Suwimonteerabutr, Fabio De Rensis

**Affiliations:** 1 Department of Obstetrics, Gynaecology and Reproduction, Faculty of Veterinary Science, Chulalongkorn University, Bangkok 10330, Thailand; 2 Centre of Excellence in Swine Reproduction, Chulalongkorn University, Bangkok 10330, Thailand; 3 Department of Veterinary Medical Science, University of Parma, Parma 43126, Italy

**Keywords:** *Andrographis paniculata*, Colostrum, Piglet Performances, Postpartum Complications, Sow

## Abstract

**Objective:**

This study evaluated the effect of *Andrographis paniculata* (*A. paniculata*) supplementation in sow diets before and after farrowing on the sow and piglets’ performances during early postpartum period and on sows’ backfat and longissimus muscle losses during lactation.

**Methods:**

Seventy Landrace×Yorkshire sows and their offspring (1,186 piglets) were distributed into three groups: control (n = 31), treatment–250 (n = 18), and treatment–1000 (n = 21). From 110.2±0.7 days of gestation until farrowing (5.8 days) and throughout the lactation period (25.2 days), sows in the control group were given the conventional lactation diet, while sows in the treatment–250 and treatment–1000 groups received supplements of 250 ppm and 1,000 ppm of *A. paniculata*, respectively.

**Results:**

In sows with parity 3–5, piglets from the treatment–1000 group had higher colostrum intake than the control and treatment–250 groups (p<0.05), but not in sows with parity 6–9. Colostrum immunoglobulin G (IgG) increased in treated sows versus controls for parity 6–9 (p<0.05), but was consistent for parity 3–5. Piglet performance until day 3 postnatal was similar across groups (p>0.05). Treatment–250 sows had higher feed intake post-farrowing than treatment–1000 sows (p<0.05). Longissimus loss was less in both treatment groups than control (p<0.05), but backfat loss was similar across groups (p>0.05). Post-partum complications were consistent across groups (p>0.05). Farrowing duration and piglet birth intervals in sows with parity 6–9 were prolonged in the treatment–1000 group.

**Conclusion:**

Supplementing with 1,000 ppm *A. paniculata* for 5.8 days pre-farrowing and 25.2 days post-farrowing enhanced sow colostrum IgG and piglet colostrum intake, while also reducing longissimus loss in sows. However, for sows of parity 6–9, this supplementation led to prolonged farrowing, increased intervals between piglet births, increased stillbirth, and reduced piglet birth weight. These effects should be considered when using *A. paniculata* supplementation.

## INTRODUCTION

From 2009 to 2019, the litter size at birth in modern hyperprolific sows used in the global swine industry increased by 4 to 5 piglets per litter [1]. However, a larger litter size led to prolonged parturition and increased the incidence of postpartum complications such as fever, postpartum dysgalactia syndrome (PDS), and diminished appetite [2]. Sows that suffer from extended farrowing duration might experience reduced appetite and insufficient feed intake during early lactation [3]. Moreover, inflammation of the uterus due to prolonged parturition can lead to a decrease in the colostrum yield of sows [4]. Therefore, a reduction in colostrum yield may decrease colostrum intake, compromising passive immunity in newborn piglets. Inadequate colostrum consumption is a primary cause of piglet pre-weaning mortality [5,6], as colostrum is crucial for energy and immunity in newborn piglets [7]. Colostrum contains immunoglobulins vital for the immune system, growth, and weight gain of piglets [8,9].

*Andrographis paniculata* (*A. paniculata*) is a medicinal plant in the Acanthaceae family. This plant is native to tropical Asia and Southeast Asia. Extracts from the plant have demonstrated antifungal, antibacterial, antiviral, antimalarial, antipyretic, anti-inflammatory, hepatoprotective, anticancer, and immunostimulatory properties [10,11]. The principal compound isolated from *A. paniculata* is andrographolide. Its anti-inflammatory mechanism involves inhibiting the production of reactive oxygen species (ROS) partly through modulation of the protein kinase C-dependent pathway, affecting the downregulation of the macrophage adhesion molecule-1 (Mac-1), which is crucial for neutrophil adhesion and transmigration [12,13]. Andrographolide also demonstrates anti-inflammatory activity by suppressing the expression of inducible nitric oxide synthase and cyclooxygenase-2 (*COX-2*) genes, through the down-regulation of nuclear factor-κB (NF-κB) and signal transducer and activator of transcription-3 (STAT3). Additionally, andrographolide exerts anti-inflammatory effects by inhibiting the expression of proinflammatory cytokines/chemokines [12,13]. In Thailand, *A. paniculata* is officially listed in the Thai herbal pharmacopoeia for treating inflammation and infections in humans, including COVID-19 [14,15].

A clinical study found that sows fed a lactation diet supplemented with 1,000 ppm of an *A. paniculata* compound weaned more piglets (+1.5 piglet per litter), had a heavier litter weight at weaning (+3.3 kg), and experienced decreased pre-weaning piglet mortality (−10.4%) [16]. Additionally, the proportion of sows with lactation problems was lower in the group treated with 1,000 ppm of an *A. paniculata* compound compared to the non-treated group (0.7% vs 5.2%, respectively) [16]. These data suggest that A. paniculata supplementation could potentially reduce postpartum illnesses in sows and decrease the severity of postpartum inflammation, fever, and pain. While it is known that the extract from *A. paniculata* has a bitter taste, a prior study observed that supplementing sow feed with an *A. paniculata* compound had no effect on feed intake during lactation [17]. Furthermore, sows receiving the *A. paniculata* compound during lactation exhibited higher feed intake than those not treated [17]. This might be attributed to the medicinal plant compound's ability to alleviate pain and fever in sows, enabling them to awaken and eat earlier, leading to increased voluntary feed intake. These findings suggest that *A. paniculata* could potentially reduce illnesses in postpartum sows and decrease the incidence of PDS. However, the impact of *A. paniculata* on sow colostrum yield and composition has not been examined, and further research is needed on its effects during the postpartum transition period in sows. Additionally, the beneficial impacts of *A. paniculata* supplementation on appetite and feed intake during hot seasons could improve the heat resistance of nursing sows and lessen the degradation of their physical condition. The objective of this study was to evaluate the effects of *A. paniculata* compound supplementation on sows in a tropical country during the hot season, specifically before farrowing and throughout lactation. The focus areas were: i) sow body condition, indicated by backfat and longissimus muscle losses, incidence of PDS, and abnormal post-parturient vaginal discharge; ii) piglet mortality within the first 3 days of postnatal life; and iii) piglet colostrum intake, sow colostrum yield, and the concentration of immunoglobulin G (IgG) in the colostrum.

## MATERIALS AND METHODS

### Animals care

The current research adhered to the principles outlined in "The Ethical Principles and Guidelines for the Utilization of Animals in Scientific Research" published by the National Research Council of Thailand. It received approval from the Institutional Animal Care and Use Committee (IACUC) in compliance with Chulalongkorn University's regulations and policies governing the treatment and utilization of experimental animals (under animal use protocol number 2031039).

### Experimental design

The present study was a case-control study that included 70 Landrace×Yorkshire crossbred sows and their offspring (n = 1,186 piglets). It was conducted in a commercial swine herd in the western region of Thailand between July and August 2020. During the experiment, the average ambient temperature was 27.1°C±1.6°C, with daily temperatures ranging from 24.4°C to 32.0°C for the lowest and highest values, respectively. The sows were divided into three groups: control (n = 31), treatment–250 (n = 18), and treatment–1000 (n = 21). From 110.2±0.7 days of gestation (ranging from 109 to 111 days) until weaning (25.2±2.6 days), sows in the control group were given the standard lactation diet, while those in the treatment–250 and treatment–1000 groups received the same diet but supplemented with *A. paniculata* compound (Herbatob-Mix; Lily FoodAnSci Co. Ltd., Samutprakarn, Thailand) at concentrations of 250 ppm and 1,000 ppm, respectively. The treatment lasted an average of 5.8±1.3 days, ranging from 3 to 10 days, prior to farrowing, and the supplement was administered continuously until weaning, which occurred 25.2±2.6 days after farrowing. The research team closely monitored the farrowing process of the sows around the clock. Detailed records related to farrowing were meticulously maintained. Postpartum characteristics of the sows, such as the occurrence of PDS, abnormal vaginal discharge, and post-parturient fever, were noted as either 'yes' or 'no'. The daily feed intake of each sow was measured for the first 3 days after farrowing. Data concerning piglet pre-weaning mortality was also gathered. Piglet colostrum intake and sow colostrum yield were estimated using an equation from Theil et al [18]. Sow colostrum IgG concentrations were determined using both enzyme-linked immunosorbent assay (ELISA) and a Brix refractometer [19].

### Animals, housing, and management

The sow parity number averaged 5.5±1.8 (ranged 3 to 9). The count of sows in parities 3, 4, 5, and 6–9 were as follows: 13, 12, 9, and 36, respectively. The gestating sows were kept in individual crates (1.25 m2) and were fed a commercial gestation diet twice daily (0700 h and 1500 h) following a standardized feeding pattern, resulting in an average of 2.5 kg per sow per day to meet or exceed their nutritional requirements [20]. Gestating sows were moved to the farrowing house at one week before the expected date of parturition. In the farrowing house, each sow was accommodated within an individual crate measuring 1.5 m^2^, positioned centrally within pens that allowed for 4.2 m^2^ of space. These pens featured fully slatted concrete flooring in the central area for the sows, with steel slats on either side of the farrowing crate to accommodate piglets. Additionally, each pen included a designated creep area for piglets, covered with a sack and occupying 0.60 m^2^ of space. During the initial week after farrowing, a heating lamp was installed in the creep area and operated during nighttime hours. Throughout the farrowing period, sows did not receive any feed. The farrowing process was closely monitored by a dedicated research team round the clock. Assistance during farrowing was only provided when dystocia was unmistakably observed. This assistance was initiated either when a 45-min interval had transpired since the birth of the last piglet, or when the sow exhibited intermittent straining along with leg paddling, or when the sow expelled fetal fluid with pronounced tail switching for 45 min without any piglet being delivered. Assisting with the birth process involved manually extracting piglets and administering 20 IU of oxytocin (CP-CIN20; L.B.S. Laboratory, Ltd., Bangkok, Thailand) when necessary. Following the completion of farrowing, sows received a regimen of antibiotics (10 mg/kg of amoxicillin trihydrate, 150 mg/mL, Amoxiguard 15% Injection LA; BIC Chemical Co., Ltd., Nakhon Pathom, Thailand) and an antipyretic medication (2 mg/kg of tolfenamic acid, 40 mg/mL; Tolfedine CS, Vetoquinol S.A., Lure Cedex, France). The initial doses of tolfenamic acid and amoxicillin trihydrate were administered one hour after farrowing and then repeated once daily for three consecutive days. Throughout the lactation period, sows were provided with two daily feedings of a lactation diet designed to meet or exceed their nutritional needs [20]. During the 2nd and 3rd weeks of lactation, sows were allowed to feed up to *ad libitum*. On average, sows consumed 6.2 kg of feed daily during these two weeks of lactation [21]. Following farrowing, the quantity of feed provided to the sows progressively increased each day, reaching a total of 7 kg after one week of lactation. Both sows and piglets had unrestricted access to water through separate nipples—one for the sow and one for the piglets. Routine procedures performed on piglets included weighing, tail docking, tooth clipping, and the intramuscular administration of a 1 mL (200 mg) iron supplement (IRON 10%; BIC Chemical Co., Ltd., Thailand) on the third day of life. Additionally, piglets received an oral coccidiocide (Toltrazuril, 50 mg/mL, 20 mg per kg, Toltrazuril 5%; Better Pharma Co., Ltd., Bangkok, Thailand) on the third day of life.

### Sow data

The data collected encompassed various sow parameters including gestation length (days), the duration of farrowing (the time span between the birth of the first and last piglets, measured in hours), the total number of piglets born in each litter (TB), the number of piglets born alive in each litter (BA), the count of stillborn piglets in each litter (SB), and the count of mummified fetuses in each litter (MF). Additionally, we recorded whether sows required assistance during birth, noting either 'yes' or 'no'. Furthermore, we assessed post farrowing complications in the sows, including the presence of fever, PDS, abnormal vaginal discharge, constipation score, and the sow's appetite on days 0, 1, 2, and 3 after parturition. Rectal temperature was determined twice a day (0700 h and 1900 h) in each sow with a digital thermometer (SOS plus Clinical digital thermometer BT-A21CN WHITE, SOS; DKSH Co. Ltd., Bangkok, Thailand). Sows with a rectal temperature of ≥39.5°C were regarded as having a fever [3]. Fever was categorized as a binary trait, with '0' indicating the absence of fever in sows and '1' indicating the presence of fever. PDS was defined based on the presence or absence of udder inflammation and/or agalactia. Udder inflammation refers to the reddening and swelling of the udder. If at least one udder exhibited signs of inflammation or agalactia, PDS was designated as '1'; otherwise, it was denoted as '0'. Abnormal vaginal discharge was defined by the presence or absence of postpartum abnormal vaginal discharge, characterized as exudates or bloody discharge ranging from dark grey to white in color. Abnormal vaginal discharge was scored as '1' when a significant amount of abnormal discharge (≥5 mL) was observed, and '0' when either no abnormal discharge was present or only a small amount of transudate (lochia) was detected. Constipation score of each sow was ranked by a visual qualitative evaluation and defined as score values ranging from 0 to 5: '1' (dry and pellet-shaped), '2' (between dry and normal), '3' (normal and soft, but firm and well formed), '4' (between normal and wet; still formed, but not firm), and '5' (very wet feces, unformed and liquid) [22]. Diminished appetite was characterized as '0' when sows exhibited a healthy appetite, consuming more than 80% of the daily feed provided, and as '1' when sows displayed indications of reduced appetite, such as decreased feed intake leading to complete anorexia [23]. Additionally, voluntary feed intake of each individual sow was collected for three days postpartum. The feed was determined for each meal from the difference between feed allowance and the remaining feed (after drying) collected one hour after the meal. Therefore, the total amount of feed intake was recorded for each sow twice a day. Feeding was provided twice a day at 0700 h and 1500 h. The daily feed intake of sow was defined as the sum of the feed intake at 0700 h and 1500 h [21].

### Piglet data

The piglet parameters recorded during the study consisted of birth order, birth interval (the time elapsed between each piglet born, min), birth weight, gender, and day of mortality. All piglets were individually identified by a pen marker on their back. The birth weight of the piglets was measured immediately after birth. The piglets were individually weighed again at 18 to 24 h of postnatal life by using a digital bench scale (Weight indicator MI-01; Linear Instrument Ltd., Nakhon Pathom, Thailand).

### Measurement of backfat thickness and longissimus muscle depth

Backfat thickness and longissimus muscle depth of the sows was measured at days 1, 14, and 21 after farrowing at P2 position on the right and left sides of the sow (at 6 cm from the midline straight above the last rib bone). Backfat thickness was measured by using A-mode ultrasonography (Renco Lean-Meater, Minneapolis, MN, USA) and longissimus muscle depth was measured by using B-mode ultrasonography (HS-2200; Honda Electronics Co. Ltd., Toyohashi, Aichi, Japan). The average between the left and the right sides was calculated. Backfat and longissimus muscle loss in each sow was defined as the difference between backfat thickness and longissimus muscle depth at days 1 and 21 after farrowing. Relative backfat loss for each sow was defined as the backfat loss (mm) divided by backfat thickness at day 1 after farrowing and multiplied by 100. The relative backfat loss was expressed as a percentage [23]. Similarly, relative longissimus muscle loss for each sow was defined as the longissimus muscle loss (mm) divided by longissimus muscle depth at day 1 after farrowing and multiplied by 100. The relative longissimus muscle loss was expressed as a percentage [24].

### Colostrum intake and yield

The formula developed by Theil et al [18] was used to estimate the individual colostrum intake for each piglet. This formula is expressed as follows: colostrum intake (g) = −106+2.26WG+200BWB+0.111D−1,414WG/D+0.0182 WG/BWB. Here, WG represents the weight gain of individual piglets from birth to 24 h after the first piglet was born (g), BWB represents the birth weight of individual piglets (kg), and D signifies the duration of colostrum intake (min). To determine the colostrum yield of the sows, we added up the colostrum intake of each individual piglet within the litter. Additionally, we calculated the colostrum intake per kilogram (kg) of body weight for piglets and used this measure for making statistical comparisons between the various groups.

### Determination of colostrum immunoglobulin G

Colostrum samples (n = 70) were collected within 3 h after birth of the first piglets to determine the Brix values shortly after collection (0.3 mL) and the remaining colostrum (5 mL) was kept at −20°C for determining colostrum IgG using ELISA. A commercial digital refractometer (Digital hand-held pocket refractometer; ATAGO, Tokyo, Japan) was used with a range of 0% to 53% Brix [19]. Colostrum samples were collected manually from all operational glands and placed in a clean bottle. Throughout the collection procedure, these samples were kept on ice within a Styrofoam container at a temperature of 4°C. Furthermore, the concentration of colostrum IgG was assessed using ELISA. The assay was carried out according to the previous study [6]. Briefly, the colostrum samples underwent centrifugation at 13,000×g for 20 min at a temperature of 4°C (utilizing the Centrifuge 5810 R from Eppendorf AG, Hamburg, Germany). Subsequently, the fat was separated and removed, leaving behind the liquid portion. This liquid was then diluted at a ratio of 1:500,000 using a sample conjugate diluent composed of 50 mM Tris buffer, 0.14 M NaCl, 1% bovine serum albumin (BSA), and 0.05% Tween 20. The concentration of IgG was assessed via ELISA, with the ELISA plate being coated with a polyclonal antibody specific to Pig-IgG (utilizing the Pig IgG ELISA kit from Bethyl Laboratories Inc., Montgomery, TX, USA). In brief, 100 μL of anti-IgG antibody was introduced into each well, followed by incubation at room temperature (25°C) for 60 min. Subsequently, the wells were washed 5 times using a washing buffer composed of 50 mM Tris buffer, 0.14 M NaCl, and 0.05% Tween 20. After this, 200 μL of a blocking solution containing 50 mM Tris buffer, 0.14 M NaCl, and 1% BSA was dispensed into each well. The mixture was then incubated at room temperature (25°C) for 30 min and subsequently washed 5 times with the washing buffer. Following the washes, 100 μL of either a standard solution or a colostrum sample was added to each well, followed by incubation at room temperature (25°C) for 60 min. The wells were once again washed five times with the washing buffer. The standard solutions encompassed IgG concentrations of 500.0, 250.0, 125.0, 62.5, 31.25, 15.6, and 7.8 g/L. All samples were subjected to duplicate analysis. Subsequently, 100 μL of horseradish peroxidase and antibody were introduced into the wells. The plates were then incubated for 60 min at room temperature and underwent five washes using the washing buffer. Following this, 100 μL of TMB (3,3′, 5,5′-tetramethylbenzidine) substrate solution was dispensed into each well and incubated in darkness at room temperature. After 15 min, the colorimetric reaction resulted in a blue product, which transitioned to yellow upon termination of the reaction by adding 100 μL of 0.18 M sulfuric acid. The absorbance was measured at 450 nm using an ELISA plate reader (Tecan Sunrise, Männedorf, Switzerland). To quantify the IgG concentration in the colostrum samples, their absorbance values were interpolated from the standard curve generated concurrently with the colostrum samples. The inter-assay and intra-assay coefficients of variation were 3.75% and 2.00%, respectively.

### Statistical analyses

The statistical analyses were performed by using SAS version 9.4 (SAS Inst. Inc., Cary, NC, USA). Descriptive statistics including number of non-missing values, general means, standard deviation (SD), and frequency analyses were conducted for all reproductive parameters. All of the continuous data were presented as mean±SD. The data were classified into two groups, i.e., sow data (n = 70) and piglet data (n = 1,186). The sow data included parity number, gestation length, farrowing duration, backfat thickness, longissimus muscle depth, TB, BA, SB, MF, and colostrum yield. The data were analyzed by using the generalized linear model procedure of SAS. The statistical models included treatment groups, parity cluster (3–5 and 6–9), and interaction between parity cluster and treatment groups. The sow was considered the experimental unit. Least-square means were obtained from each class of the factors and were compared using the least significant difference test.

The piglet data included birth interval, birth weight, body weight at 24 h after birth, colostrum intake, and colostrum intake per kg of body weight of piglet. The piglet data were analyzed by using the generalized linear mixed model (MIXED) procedure of SAS. The statistical models included treatment groups, parity cluster (3–5 and 6–9), and interaction between parity cluster and treatment groups. The sow identity was included in the statistical model as a random effect to adjust for the maternal effect. The piglet was considered the experimental unit. Least-square means were obtained from each class of the factors and were compared using the least significant difference test.

The postpartum complications index, which comprised variables such as fever (present or absent), PDS (present or absent), abnormal vaginal discharge (present or absent), and constipation (categorized as constipation with a score of 0 to 2 and non-constipation with a score of 3 to 5), was expressed as percentages. These indices were then subjected to comparison using logistic regression across three groups: sows that received a lactation diet with no *A. paniculata* compound supplementation (control), those with 250 ppm supplementation (treatment–250), and those with 1,000 ppm supplementation (treatment–1000). The data were analyzed by using the generalized linear mixed model procedure (GLIMMIX) of SAS. The statistical models included treatment groups, parity cluster (3–5 and 6–9), and interaction between parity cluster and treatment groups. The sow was considered the experimental unit. Least-square means were obtained from each class of the factors and were compared using the least significant difference test. Furthermore, Pearson’s correlation analysis was performed to assess the connection between sow feed intake and their colostrum yield. This correlation analysis was carried out separately for each parity and within different treatment groups. For all statistical tests, p<0.05 was considered to be statistically significant.

## RESULTS

### Descriptive data

Table 1 presents descriptive statistics on the reproductive performance of sows segmented by parity clusters. Across different parities, the observed values for TB, BA, SB, and MF were 16.7±3.2, 14.5±2.9, 1.6±1.9, and 0.6±0.9, respectively. The average gestation length was 116.1±1.1 days, and the duration of farrowing averaged 5.8±4.7 h. The mean backfat thickness for sows was 14.6±3.1 mm at 1 day after farrowing and 12.7±2.8 mm at 21 days post-farrowing. During the lactation period, sows experienced an average reduction in backfat of 11.7%. For parity numbers 3–5 and 6–9, the relative back fat loss (%) was −14.9±11.2 and −8.6±11.9, respectively (p = 0.009). The brix value of sows with parity numbers 6–9 was higher than sows with parity numbers 3–5 (26.0%±2.5% and 27.6%±3.4%, respectively, p = 0.036). Additionally, the average depth of the longissimus muscle in sows on day 1 and day 21 postpartum was 41.2±5.1 mm and 32.2±3.6 mm, respectively. The loss of longissimus muscle during the lactation period averaged 20.9%. The average colostrum yield and colostrum IgG concentration were 5.5±1.1 kg and 44.1±12.7 g/L, respectively. Colostrum yield was higher on average for sows with parity numbers 3–5 compared to those with parity numbers 6–9 (5.9±1.0 kg vs 5.1±1.1 kg, respectively, p = 0.005) (Table 1).

Of the total offspring (n = 1,186), 1,030 piglets (86.9%) were born alive, 114 piglets (9.6%) were stillborn, and 42 piglets (3.5%) were mummified fetuses. The average time between births was 12.1 min, and newborn piglets weighed, on average, 1,285±322 g. The mortality rate for piglets 3 days postpartum stood at 11.4%. Piglets that did not survive beyond one week had an average age of 2.3±1.3 days. Moreover, the average colostrum intake by piglets was 405±154 g.

Table 1 contrasts piglet characteristics between sows of parities 3–5 and 6–9. Piglets from sows of parities 6–9 had, on average, longer birth intervals than those from sows of parities 3–5. However, most other piglet characteristics did not significantly differ between the two parity groups. Yet, when considering treatment effects, the birth interval in the treatment–1000 group for parity 6–9 was notably longer (with intervals of 18.1±3.4, 17.0±3.9, and 33.3±3.7 for the control, treatment–250, and treatment–1000 groups, respectively). Also, there was a reduction in piglet birth weight (with weights of 1,327±27.9, 1,296±25.8, and 1,245±26.0 for the control, treatment–250, and treatment–1000 groups, respectively).

### Effect of *A. paniculata* on sows performances

**Figure 1:**
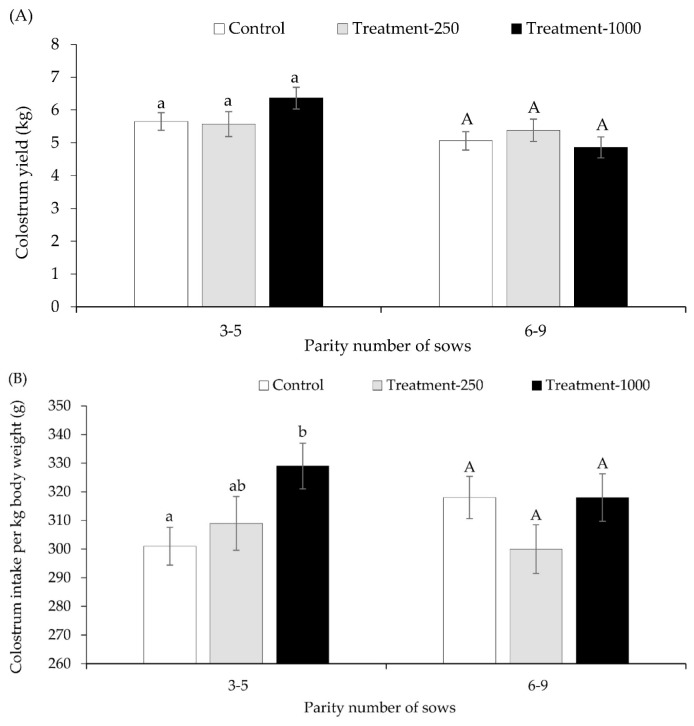
(A) Colostrum yield of sows and (B) colostrum intake of piglets per kg of body weight in control (n = 31), treatment–250 (n = 18), and treatment–1000 (n = 21) groups, respectively. Comparation between sows with parity 3–5 and 6–9. Letters that are the same within parity groups indicate significant difference (p>0.05), whereas different letters indicate significant differences (p<0.05).

Table 2 presents data on farrowing duration, colostrum production, colostrum IgG levels, and sow feed intake across three groups: control, treatment–250, and treatment–1000, sorted by their parity clusters. Notably, sows of parities 6–9 had a longer farrowing duration than those of parities 3–5 (Table 1). Moreover, the colostrum IgG concentration in the treatment–1000 group was significantly higher than in both the control and treatment–250 groups, showing values of 50.2±2.6 g/L compared to 41.5±2.0 g/L and 40.6±2.7 g/L, respectively (p<0.05) (Table 2). In all groups, sows of parities 3–6 had a higher colostrum yield than those of parities 6–9 (Table 1). Yet, no difference in colostrum yield between the control and treatment groups was observed for either parities 3–5 or 6–9 (Figure 1A).

During lactation, sows with a parity number of 6–9 in the treatment–1000 group experienced less longissimus muscle loss (–15.9%±3.3%) compared to the control group (−27.9%± 2.9%, p = 0.009). This loss was not significantly different from the treatment–250 group (−23.1%±3.5%, p = 0.152) as shown in Table 2. Backfat loss was similar across all groups for sows with parities of 3–5 and 6–9 (Table 2). Furthermore, there were no observed differences in postpartum complications between the treatment and control groups.

### Effect of *A. paniculata* on piglet performances

Table 3 details the piglets' performance across three groups: the control, treatment–250, and treatment–1000, further divided by their respective parity clusters. On the third day, the piglet mortality rates among these groups did not differ significantly: 9.1% for the control, 13.1% for both treatment −250 and treatment–1000 (p = 0.143). Figure 1B elaborates on the colostrum intake per kg of piglet body weight for the three groups within sows of parities 3–5 and 6–9. Regardless of parity, piglets in the treatment–1000 group consumed more colostrum per kg of body weight than those in both the control and treatment–250 groups, with respective values of 324±5.8 g, 309±4.9 g, and 305±6.3 g (p<0.05). Notably, in the treatment–1000 group, piglets from sows of parities 3–5 had an intake of 329±8.0 g per kg, higher than the control's 301±6.6 g (p = 0.007). However, this intake did not significantly differ from the treatment–250 group's 309±9.4 g (p = 0.108) (Figure 1B). In the sows of parity groups 6–9, no discernible difference in colostrum intake per kg was noted across the treatment groups (p> 0.05) (Figure 1B). Moreover, piglets in the treatment–1000 group under sows of parities 3–5 saw a numerical increase in weight gain within the first 24 h postpartum (93.0±8.3 g) when compared to the control (76.2±6.8 g, p = 0.117) and treatment–250 (73.4±9.7 g, p = 0.126).

### Effect of *A. paniculata* on feed intake and postpartum complications of sows

In sows with parity numbers 6–9, the treatment–1000 group demonstrated a lower daily feed intake in the first 3 days post-farrowing compared to both the treatment–250 and control groups (Table 2). Yet, this difference was not evident in sows with parity numbers 3–5 (Table 2). Across all treatment groups and sow parity numbers, there was an observed trend indicating a positive correlation between sow feed intake and colostrum yield (r = 0.221, p = 0.065). The correlation analysis, segmented by both parity and treatment groups, indicated that parity did not significantly influence the relationship between colostrum yield and feed intake (p>0.05). However, the correlation between colostrum yield and feed intake varied among the treatment groups. In the treatment–1000 group, a positive correlation was evident (r = 0.509, p = 0.018), whereas in the control and treatment–250 groups, no such relationship was identified (p>0.05). The occurrence of post-parturient complications, including fever, PDS, abnormal vaginal discharge, and constipation score, did not differ significantly among the control, treatment–250, and treatment–1000 groups (p>0.05).

## DISCUSSION

### Farrowing duration and postpartum complication

The effective management of the sow's farrowing process is crucial for enhancing piglet survival, particularly in highly prolific sows. Hence, understanding factors related to the duration of farrowing is significant. In this study, the sows exhibited an average farrowing duration (stage II of parturition) of 5.8 h, which is lengthier compared to earlier research conducted in Thailand [25,26]. Additionally, the average total number of piglets born per litter in this study (16.7 TB) surpassed the earlier reports from a decade ago in tropical environments like Thailand, showing an increase of +5.4 piglets per litter (11.3 TB) [27]. These findings indicate that the sows in the present commercial herd possess hyperprolific sow genetics. Consequently, effective prepartum and postpartum care have become even more crucial than in the past. In this study, the duration of farrowing for sows within parity 3–5 did not differ significantly between the control group and the treatment–250 and treatment–1000 groups. Conversely, sows within parity 6–9 in the treatment–1000 group experienced a notably prolonged farrowing duration compared to the other groups, averaging at 9.8 h. This elongation in farrowing duration could potentially be attributed to this group having a larger litter size (17.2 TB) and a higher count of stillborn piglets (3.4 piglets per litter) compared to the other groups.

Sows having parity numbers from 6 to 9 and treated with 1,000 ppm of *A. paniculata* showed longer farrowing durations (Table 2), increased birth intervals (Table 3), a higher number of stillborn piglets (Table 2), and lower piglet birth weights (Table 3). Therefore, administering a high dose of *A. paniculata* in older sows led to a notable decrease in the number of piglets born alive, as compared with those given a lower dose (Table 2). Furthermore, a numerical increase in piglet mortality was observed by the third day postpartum, though it was not statistically significant. These negative effects might be attributed to the bitter taste of *A. paniculata*, possibly reducing the sows' daily feed intake before farrowing. While we do not have data on this specific feed intake, the bitterness of *A. paniculata* could deter appetite and lead to reduced consumption. However, its influence on the neuroendocrine system governing farrowing merits consideration. A previous study indicated that the bitter taste of *A. paniculata* did not impact lactation feed intake [17]. Nonetheless, both colostrum yield and backfat loss in this group were comparable to other groups. Also, the treatment showed no noticeable effects on postpartum complications such as fever, PDS, vaginal discharge, and constipation on days 1 through 3.

### Feed intake of sow

Our study indicates that average daily feed intake during the first 3 days after farrowing of sows in the treatment–250 group was higher than sows in the treatment–1000 group. However, the decrease in daily feed intake among sows treated with 1,000 ppm *A. paniculata* was evident in sows with a parity number of 6–9, while it was not observed in sows with a parity number of 3–5. These findings indicate that, in sows with parity numbers 3–5, the supplementation of 1,000 ppm of this compound in the feed of lactating sows did not compromise voluntary feed intake as compared to the control group, but the supplement of 250 ppm of *A. paniculata* can improve feed intake of sows during the first 3 days post farrowing. Thus, a low dose of *A. paniculata* may be able to improve voluntary feed intake of multiparous sows, while the high dose of *A. paniculata* may interfere the palatability of the feed and may not have any a significant benefit on feed intake of the sow. The reason might be due to *A. paniculata* is rather bitter. Furthermore, the decreased feed intake observed in sows with parity numbers 6–9 who were administered 1,000 ppm of *A. paniculata* suggests that these older sows might have a heightened sensitivity to bitter tastes compared to younger sows. Consequently, the addition of 1,000 ppm of *A. paniculata* to the feed of sows with parity numbers exceeding 6 should either be avoided or carried out with caution. A prior investigation has shown that adding cinnamon oil to the diet can enhance feed consumption in multiparous sows, while it does not have the same effect in primiparous sows [21]. This implies that sows' perception of feed palatability might be affected by age or parity number, potentially leading to unfavorable results.

### Colostrum yield and immunoglobulin G concentration

Preweaning mortality in piglets is a major economic and welfare issue in commercial pig production worldwide [28]. Earlier studies have reported that the preweaning mortality rate of live-born piglets in a commercial swine herd range from 11.0% to 24.0% [4,29,30]. On average, the piglet preweaning mortality in the present study was 6.0% and 11.4% at 1 and 3 days of postnatal life, respectively and there were no differences on piglet mortality during the first 3 days of life among groups. The major causes of piglets that die before weaning include low birth weight, insufficient colostrum intake, low body temperature, and low blood glucose [9,28]. Due to the epitheliochorial nature of the porcine placenta, piglets are dependent on obtaining maternal immunoglobulins through colostrum ingestion after birth [31]. Deceased piglets tend to have reduced serum IgG concentrations compared to their surviving counterparts, indicating inadequate colostrum intake [32].

*A. paniculata* compound has anti-inflammatory, antipyretic, and pain relief properties [11,12]. These properties may be able to reduce stress and enhance colostrogenesis in sows during transition period. Thus, information concerning IgG concentration in colostrum is important for field practice to reduce preweaning mortality of piglets. In fact, the transfer IgG from the colostrum to piglet blood provides passive immunity to the offspring [31,32]. The present study revealed that treatment of 1,000 ppm of *A. paniculata* in prepartum sows increased colostrum IgG concentration. This finding expands previous clinical observation that the supplementation of an *A. paniculata* compound with 1,000 ppm in lactation diet improve the number of piglets at weaning, litter weight gain and piglet preweaning mortality [16]. The present study found that the supplementation of 1,000 ppm of *A. paniculata* compound increased colostrum IgG concentration but the supplementation of 250 ppm *A. paniculata* did not. This indicates that the effect of *A. paniculata* on IgG concentration is dose dependent.

The colostrum yield and colostrum IgG concentration in sows can exhibit significant variability among individual animals, as reported in previous studies [8,33]. In our current investigation, the average colostrum yield from sows was 5.5±1.1 kg, and there were no discernible differences between the various groups. This lack of distinction may be attributed to the fact that the colostrum yield at this particular farm is already considered quite satisfactory, as evidenced by previous research [8,34,35]. Consequently, it could be challenging to observe further improvements in colostrum yield in this context. It would be of particular interest to assess the treatment's effectiveness in a farm characterized by a lower colostrum yield.

### Colostrum intake

Inadequate intake of colostrum is the primary cause piglet mortality during the first few days of postnatal life [8]. Colostrum intake depends on the ability of sows to produce colostrum and the ability of piglets to extract, suckle, and ingest the colostrum [36]. In Thailand, 26.6% of the live-born piglets have inadequate colostrum intake during the first 24 h of life [37]. Factors negatively affect the piglet colostrum intake include TB, BA, SB, and cumulative birth interval [37]. On the other hand, factors positively associated with the piglet colostrum intake include body weight at birth, body weight gain, and rectal temperature at 24 h of postnatal life [37]. Furthermore, the percentage of piglets lacking sufficient colostrum in a litter increases from 13.2% to 33.1% when the litter size expands from 7–13 to ≥18 piglets per litter [37]. Therefore, when the modern hyperprolific sow genetics has been introduced to the swine industry, strategies to enhance colostrum intake of piglets should be highly concerned. In the present study, piglets in the treatment–1000 group consumed a greater amount of colostrum per kg of body weight compared to those in the control and treatment–250 groups. This could be attributed to the anti-pyretic and anti-inflammatory properties of *A. paniculata*, which potentially alleviate post farrowing pain in treated sows and enhance their postpartum comfort. As a result, this enhanced comfort could potentially improve the lactation capacity of the treated sow. Furthermore, this study revealed that sows with parity numbers 3–5 had a colostrum yield that was 0.8 kg higher compared to sows with parity numbers 6–9. This highlights the impact of aging on a sow's ability to produce colostrum. To our knowledge, this is the initial report underscoring the influence of aging in sows on colostrum production. In a previous study, it was found that sows with parity numbers 2–4 exhibited a higher colostrum yield compared to both primiparous sows and those with parity numbers 5–6 [38]. However, data regarding colostrum yield in sows with parity numbers above 6 have not been previously documented. These findings emphasize that inadequate colostrum production could potentially be another factor affecting the culling decisions in commercial swine herds. Furthermore, when devising strategies involving feed additives to enhance colostrum production in sows, the factor of sow aging should be considered. Moreover, within the scope of our current investigation, the supplementation of 1,000 ppm of *A. paniculata* did not result in an enhancement of body weight gain during the initial 24 h following birth, despite an observed increase in colostrum intake per kg of body weight. This discrepancy may be attributed to the fact that piglet weight gain is commonly used as a primary indicator for estimating colostrum consumption in sows. Nevertheless, this metric does not account for the piglets' initial birth weight or the timing of colostrum consumption. It is noteworthy, however, that although no statistically significant difference was detected, it is worth highlighting that piglets in the treatment–1000 group, under sows with parities 3–5, exhibited a numerical increase in weight gain during the first 24 h postpartum (93.0±8.3 g), in contrast to both the control group (76.2±6.8 g) and the treatment–250 group (73.4±9.7 g).

### Sow backfat and longissimus muscle loss

In this study, sows receiving a lactational diet supplemented with 1,000 ppm of *A. paniculata* compound showed significantly reduced relative longissimus muscle depth losses in the 6–9 parity group compared to the control. However, there were no noticeable differences when compared to the treatment–250 group. These data suggest that the sows treated with 1,000 ppm of *A. paniculata* might have had an increased feed intake. The dosage of the supplement, whether 250 ppm or 1,000 ppm, or its potential bitter taste, does not seem to influence this, as both treatment groups exhibited similar outcomes (14.7% for treatment–250 and 14.8% for treatment–1000). In terms of parity, no differences in longissimus losses were observed. Although there is no prior documentation on the effect of *A. paniculata* on protein metabolism, the observed trend merits further research due to its potential significant influence on post-weaning fertility performances. No differences in backfat losses were discernible across treatments or parities. Considering all animals in the study had acceptable back fat levels (14.6±3.1 mm at lactation's onset) [39], pinpointing a difference could be difficult. The observations related to longissimus muscle and backfat thickness are consistent with a recent study by Costermans et al [40], wherein feed restriction resulted in a 70% higher lactational body weight loss and a 76% greater longissimus muscle depth loss, yet backfat loss remained comparable to fully fed sows.

## CONCLUSION

Administering either a 1,000 ppm or a 250 ppm *A. paniculata* compound to sows for 31 days, which included 5.8 days before farrowing and 25.2 days during the lactation period, led to an increase in colostrum IgG concentration and a rise in colostrum intake per kilogram of body weight in piglets born to sows with parities of 3–5. However, piglet performance remained unchanged in the initial 3 days postpartum. In sows with parity numbers 6–9, however, the number of stillborn piglets was increased in those treated with 1,000 ppm *A. paniculata*. Moreover, no changes were observed in postpartum complications such as fever, PDS, vaginal discharge, and constipation. Intriguingly, while the treatment did not impact backfat, there was a decrease in the longissimus muscle loss percentage in *A. paniculata*-treated sows. This finding suggests that the treatment might have a positive effect on fertility post-weaning. Yet, it is vital to acknowledge the observed impact of using 1,000 ppm *A. paniculata* supplementation in old sows, which includes extending the farrowing duration, increasing piglet birth intervals, raising stillbirth rates, reducing the number of piglets born alive, and decreasing piglet birth weight.

## Figures and Tables

**Table 1:** Reproductive outcomes of sows and characteristics of piglets categorized by parity classes

Variables	Parity numbers		p-value

	3 to 5	6 to 9	
Sows			
Number of observations	34	36	
Parity number	3.9±0.8	6.9±0.9	<0.001
Gestation length (days)	115.9±1.0	116.3±1.2	0.148
Farrowing duration (h)	5.2±4.1	6.4±5.1	0.300
Total number of piglets born per litter	16.8±3.2	16.7±3.2	0.897
Number of piglets born alive per litter	15.4±2.8	13.7±2.9	0.015
Number of stillborn piglets per litter	0.8±1.1	2.4±2.3	<0.001
Number of mummified fetuses per litter	0.6±0.8	0.6±0.9	0.915
Backfat thickness at 1 day after farrowing (mm)	14.8±3.5	14.3±2.8	0.494
Backfat thickness at 14 days after farrowing (mm)	13.5±2.5	13.5±2.9	0.983
Backfat thickness at 21 days after farrowing (mm)	12.4±2.5	13.1±3.0	0.334
Longissimus muscle depth at 1 day after farrowing (mm)	40.8±5.5	41.6±4.8	0.550
Longissimus muscle depth at 14 days after farrowing (mm)	37.9±3.6	36.9±4.7	0.329
Longissimus muscle depth at 21 days after farrowing (mm)	32.6±3.8	31.7±3.3	0.316
Relative backfat loss (%)	−14.9±11.2	−8.6±11.9	0.009
Relative longissimus muscle loss (%)	−18.9±13.5	−22.9±10.2	0.164
Immunoglobulin G concentration in colostrum (g/L)	41.3±10.8	46.7±13.8	0.076
Brix value (%)	26.0±2.5	27.6±3.4	0.036
Colostrum yield (kg)	5.9±1.0	5.1±1.1	0.005
Piglets			
Number of observations	571	615	
Birth interval (min)	16.6±33.4	22.8±65.6	0.042
Cumulative birth interval (min)	129.8±105.4	140.0± 155.9	0.192
Birth weight (g)	1,279±324	1,292±320	0.514
Weight at day 1 (g)	1,375±359	1,384±352	0.683
Weight at day 3 (g)	1,638±267	1,610±386	0.184
Duration of colostrum suckling (min)	1,349±126	1,385±101	<0.001
Piglet weight gain during the first 24 h postpartum (g)	80.8±107.9	79.6±97.1	0.860
Colostrum intake (g)	403±158	384±151	0.584
Colostrum intake per kg body weight (g)	311±109	312±88	0.857
Day of mortality (d)	2.3±1.2	2.2±1.5	0.786
Preweaning mortality at d 3 (%)	11.6	11.1	0.772

Mean±standard deviation.

**Table 2:** Performance indicators for sows (farrowing traits, colostrum production, backfat loss, and longissimus muscle loss), colostrum IgG levels, and average daily feed intake in the control, treatment–250, and treatment–1,000 groups categorized by parity cluster (least square means±SEM)

Variables	Group		

	Control	Treatment–250	Treatment–1000
Parity numbers 3–5			
Colostrum yield (kg)	5.65±0.27	5.57±0.38	6.37±0.33
Total number of piglets born per litter	16.4±0.8^a^	15.5±1.1^ab^	18.4±0.9^b^
Number of piglets born alive per litter	14.9±0.7	14.8±0.9	16.8±0.8
Number of stillborn piglets per litter	0.8±0.4	0.3±0.6	1.2±0.6
Number of mummified fetuses per litter	0.8±0.2	0.5±0.3	0.4±0.3
Farrowing duration (min)	306±66	241±93	376±84
Farrow assistance (%)	18.7	25.0	30.0
Relative backfat loss (%)	−12.2±2.9	−19.4±4.1	−15.7±3.7
Relative longissimus muscle loss (%)	−23.5±2.8	−14.7±3.9	14.8±3.5
Colostrum IgG (g/L)	42.6±2.8	37.6±4.0	42.4±3.8
Brix (%)	25.6±0.7	26.9±1.1	25.8±0.9
Daily feed intake during the first 3 days after farrowing (kg/d)	3.37±0.09	3.58±0.13	3.59±0.18
Parity numbers 6–9			
Colostrum yield (kg)	5.06±0.28	5.38±0.34	4.86±0.32
Total number of piglets born per litter	15.5±0.8	17.9±0.9	17.2±0.9
Number of piglets born alive per litter	13.1±0.7^a^	15.7±0.8^b^	12.8±0.8^a^
Number of stillborn piglets per litter	2.1±0.5^ab^	1.7±0.6^a^	3.4±0.5^b^
Number of mummified fetuses per litter	0.4±0.2	0.5±0.3	1.0±0.3
Farrowing duration (min)	282±68^a^	301±84^a^	589±84^b^
Farrow assistance (%)	33.3	10.0	36.4
Relative backfat loss (%)	−8.5±3.0	−8.0±3.7	−9.4±3.5
Relative longissimus muscle loss (%)	−27.9±2.9^a^	−23.1±3.5^ab^	−15.9±3.3^b^
Colostrum IgG (g/L)	40.5±2.9^a^	43.6±3.6^a^	58.1±3.4^b^
Brix (%)	27.1±0.8	28.9±0.9	26.9±0.9
Daily feed intake during the first 3 days after farrowing (kg/d)	3.53±0.09^ab^	3.54±0.12^a^	2.98±0.11^b^

Ig, immunoglobulin G; SEM, standard error of the mean.

a,b Different superscripts within row differ significantly (p<0.05).

**Table 3:** Traits of piglets within the control, treatment–250, and treatment–1000 groups based on parity clusters (least square means±standard error of the mean)

Variables	Group		

	Control	Treatment–250	Treatment–1000
Parity numbers 3–5			
Piglet mortality at day 3 (%)	9.2	13.6	13.6
Colostrum intake (g)	395±10.2	402±14.6	416±12.4
Colostrum intake per kg body weight (g)	301±6.5^a^	309±9.4^ab^	329±8.0^b^
Birth interval (min)	18.6±3.2	15.5±4.7	14.3±3.9
Birth weight (g)	1,299±20.9	1,282±29.6	1,250±24.9
Weight at day 1 (g)	1,389±23.4	1,372±33.7	1,355±28.7
Piglet weight gain (g)	76.2±6.8	73.4±9.7	93.0±8.3
Parity numbers 6–9			
Piglet mortality at day 3 (%)	8.7	12.7	12.4
Colostrum intake (g)	422±11.5	393±13.1	406±12.9
Colostrum intake per kg body weight (g)	318±7.4	300±8.4	318±8.3
Birth interval (min)	18.1±3.4^a^	17.0±3.9^a^	33.3±3.7^b^
Birth weight (g)	1,327±27.9^a^	1,296±25.8^ab^	1,245±26.0^b^
Weight at day 1 (g)	1,416±26.4	1,381±30.2	1,347±29.7
Piglet weight gain during the first 24 h postpartum (g)	87.5±7.6	68.6±8.8	80.2±8.6

a,b Letters within parity groups signify no significant difference (p>0.05), while differing letters represent significant differences (p<0.05).
